# Is therapeutic hypothermia immunosuppressive?

**DOI:** 10.1186/cc11266

**Published:** 2012-06-07

**Authors:** Kees H Polderman

**Affiliations:** 1 Department of Critical Care Medicine, University of Pittsburgh Medical Center, Pittsburgh, PA, USA

## Introduction

The answer to the question in the title of this article would appear obvious. Even the term 'to catch a cold' is partly based on the widely-held assumption that lower temperatures or a chill can decrease the resistance to certain viral infections such as the common cold [1]. Furthermore, a number of studies have clearly demonstrated that inadvertent decreases in temperature in the perioperative setting, and in some other situations, can significantly increase infection risk.

However, the question is not as easy to answer in cases where hypothermia is applied deliberately and with various precautions, which is the case in therapeutic cooling. In these cases potentially harmful responses such as shivering and tachycardia are carefully controlled, body temperatures are not allowed to drop below a predefined setpoint, and every effort is made to prevent side effects [2,3]. Moreover, even the evidence that hypothermia plays a role in the development of the common cold is mixed, and the few studies performed to address this issue did not support the popular belief that colds are associated with exposure to a cold environment [1,4].

The relationship between hypothermia and immune function are briefly discussed below.

### *In vitro *studies and animal experiments

In most types of acute brain injury there is a significant and protracted inflammatory response in the hours following the acute event, whether this is ischemic, traumatic, or a combination of both. Proinflammatory mediators such as TNFα and IL-1 are released in large quantities by astrocytes, microglia, and endothelial cells following an episode of ischemia and reperfusion [2]. The levels of these mediators begin to rise around 1 hour after injury and remain elevated for up to 5 days [2,5,6]. This in turn stimulates the chemotaxis of activated leucocytes across the blood-brain barrier and leads to an accumulation of inflammatory cells in the injured brain, as well as the emergence of adhesion molecules on leukocytes and endothelial cells. Simultaneously activation of the complement system occurs, beginning in the very early stages after brain injury. This further stimulates the passage of neutrophils and (in later stages) monocytes/macrophages [6]. These inflammatory and immunological responses occur especially during reperfusion and are accompanied by free radical production (see below). These inflammatory responses can cause significant (additional) injury through the phagocytic actions of macrophages, synthesis of toxic products, and further stimulation of immune reactions in a vicious cycle.

Thus it can be argued that, at least in the initial stages of acute brain injury, a hyperinflammatory state exists. As explained in some more detail below hypothermia attenuates this proinflammatory state, but this in itself does not constitute immune suppression. On the other hand, it should be realized that the proinflammatory response outlined above is to some extent physiological; there is evidence suggesting that some inflammatory mediators have neuroprotective properties, although many others are neurotoxic [6-9]. Thus attenuating the response could have protective effects, but also some detrimental ones [2,8,9].

On balance there is persuasive evidence suggesting that the production of cytokines and leukocyte infiltration is disproportionate and harmful, and can significantly increase the risk and extent of brain cell injury and infarction [6-15]. Especially, the IL-1 family appears to be important in this regard [15]. The destructive aspects of inflammation appear to outweigh the potential benefits especially in the later stages of injury [2,6-9]. Thus there is a potential time window for therapeutic interventions to block or mitigate this process before it causes permanent injury.

Many animal experiments and *in vitro *studies have shown that mild hypothermia can suppress harmful inflammatory reactions that damage potentially viable nerve cells and astrocytes [10-12]. Hypothermia can also decrease production of leukotrienes and nitric oxide, prevent reperfusion-related DNA injury and lipid peroxidation, and impair neutrophil and macrophage function [2,3]. Thus on balance the protective effects of cooling are likely to outweigh the potential negative effects. However, this does not mean that there are no negative effects, especially if the temperature drops below 32°C. It should be kept in mind that the suppression of inflammatory responses will occur in all organs, not just the injured ones; this is one of the reasons why inhibition of the immune response can lead to increased infection risk. Moreover, the effect is present regardless of whether a local or general hyperinflammatory state exists.

The systemic effect on the immune system can be enhanced by a decrease in the white blood cell count, which can begin at temperatures below 32 to 33°C (although major decreases usually occur only at temperatures below 30 to 31°C). Biggar and coworkers reported a drop in WBC count from 6.0 ± 0.6 to 2.3 ± 0.3 when hypothermia of 29°C was induced in pigs [16]. They did not observe significant changes in the number of circulating mature or immature neutrophils, and reported that neutrophil demargination after administration of intravenous catecholamines was similar at 37°C and 29°C. However, they also observed that administration of corticosteroids and, importantly, of endotoxins failed to stimulate the normal release of neutrophils from the bone marrow [16]. Failure of this response could significantly impact infection risks [2].

Most of these observations were made in animal experiments, but the hypothermia-induced suppression of the hyperinflammatory responses and decrease in WBC count have been confirmed in clinical reports in humans with traumatic brain injury [10,11,17].

Thus the very mechanisms that provide tissue protection could simultaneously impair the patients' ability to fight infections. In addition, lowering body temperature can lead to a decrease in insulin secretion and to induction of insulin resistance [2,3]. This can lead to hyperglycaemia, which can in turn impair leucocyte function and further increase infection risks. Finally, hypothermia can cause vasoconstriction in the skin, which could increase the risk for bedsores and surgical wound infections.

In summary, based on the *in vitro *evidence we would expect hypothermia to inhibit the mostly harmful neuroinflammatory response and ameliorate the hyperinflammatory state that occurs after acute injury, but at the price of increasing infection risk.

### Clinical evidence

Clinical studies reporting the infection risks associated with therapeutic cooling in different categories of patients with acute brain injury have produced divergent results; studies in patients who develop *accidental *hypothermia have mostly reported higher infection risks.

The link between accidental hypothermia in the perioperative setting and a higher incidence of surgical wound infections was first demonstrated by Kurz and coworkers in 1996 [18], and has since been confirmed in numerous studies in various categories of surgical patients [19-26]. The most recent example is a study by Seamon and coworkers, who found that intraoperative hypothermia (below 35°C) was independently associated with surgical site infection rates after trauma laparotomy [26]. Local factors such as hypothermia-induced vasoconstriction in the skin may add to the underlying immunosuppressive effect to further increase the rate of wound infections. Recently, Laupland and colleagues reported that severe hypothermia (<32°C, but not 32 to 35.9°C) was associated with significant increases in risk for infections acquired in ICU [27].

The link between hypothermia and infections is far less clear when mild hypothermia is induced under controlled circumstances. Numerous studies in patients with post-hypoxic brain injury following cardiac arrest have not reported significant increases in rates of infections, although some have reported trends in that direction [28]. Seven multicentered trials in newborn babies treated with neonatal asphyxia for 48 to 72 hours also did not report consistent increases in infection risks [28].

In contrast, clinical studies in patients with ischemic stroke and TBI have tended to find higher risks of infection, especially pneumonia, in patients treated with hypothermia. For example, Hemmen and coworkers reported a rate of pneumonia of 50% in patients with ischemic stroke treated with hypothermia and thrombolysis, compared to 10% in controls [29]. Although the overall outcome was better in hypothermia patients in spite of the high infection rate, this indicates that use of hypothermia in these patients may present significant difficulties.

Some studies using hypothermia in patients with severe traumatic brain injury have also reported high infection rates [28]. There is evidence that this can be prevented by a combination of preventive measures, perhaps including use of antibiotic prophylaxis such as selective decontamination of the digestive tract (SDD) [28,30,31].

In one example, Kamps and coworkers reported on their use of prolonged therapeutic cooling to control intracranial pressure in patients with severe traumatic brain injury, in a setting where SDD was routinely used, and reported that infection rates were 20% in patients treated with hypothermia and 34.4% for matched controls [31]. Most notably, the risk of ventilator-associated pneumonia was the same in patients treated with hypothermia compared with matched controls.

## Conclusion

Hypothermia impairs immune function and inhibits various inflammatory responses. This is inherent to the treatment, and impairment of harmful inflammatory reactions in the brain may be one of the mechanisms through which hypothermia can exert protective effects. Hypothermia-induced insulin resistance and hyperglycemia may further increase infection risks. In clinical studies, hypothermia has been most clearly linked to infection risk in the context of accidental hypothermia; controlled therapeutic cooling appears to carry a lower risk, especially if hypothermia is used for limited periods of time (<48 hours). The risk appears to increase with prolonged use, and careful monitoring is required in these patients. Prophylactic antibiotics may be considered in high-risk patients who are cooled for prolonged periods.
